# Quality by Design (QbD) Approach to Develop Colon-Specific Ketoprofen Hot-Melt Extruded Pellets: Impact of Eudragit^®^ S 100 Coating on the In Vitro Drug Release

**DOI:** 10.3390/pharmaceutics16101265

**Published:** 2024-09-27

**Authors:** Sateesh Kumar Vemula, Sagar Narala, Prateek Uttreja, Nagarjuna Narala, Bhaskar Daravath, Chamundeswara Srinivasa Akash Kalla, Srikanth Baisa, Siva Ram Munnangi, Naveen Chella, Michael A. Repka

**Affiliations:** 1 Department of Pharmaceutics and Drug Delivery, School of Pharmacy, The University of Mississippi, University, MS 38677, USAputtreja@go.olemiss.edu (P.U.);; 2 Department of Pharmaceutics, School of Pharmaceutical Sciences, Lovely Professional University, Phagwara 144411, Punjab, India; 3 Department of Pharmaceutics, GITAM School of Pharmacy, GITAM Deemed to be University, Rudraram, Patancheru, Sangareddy, Hyderabad 502329, Telangana, India; bdaravat@gitam.edu; 4 Department of Pharmaceutical Technology (Formulations), National Institute of Pharmaceutical Education and Research (NIPER), Guwahati 781101, Assam, India; 5 Pii Center for Pharmaceutical Technology, The University of Mississippi, University, MS 38677, USA

**Keywords:** Box–Behnken Design, colon specific, enzyme-triggered, hot-melt extrusion, pH-sensitive

## Abstract

Background: A pelletizer paired with hot-melt extrusion technology (HME) was used to develop colon-targeted pellets for ketoprofen (KTP). Thermal stability and side effects in the upper gastrointestinal tract made ketoprofen more suitable for this work. Methods: The pellets were prepared using the enzyme-triggered polymer Pectin LM in the presence of HPMC HME 4M, followed by pH-dependent Eudragit^®^ S 100 coating to accommodate the maximum drug release in the colon by minimizing drug release in the upper gastrointestinal tract (GIT). Box–Behnken Design (BBD) was used for response surface optimization of the proportion of different independent variables like Pectin LM (A), HPMC HME 4M (B), and Eudragit^®^ S 100 (C) required to lower the early drug release in upper GIT and to extend the drug release in the colon. Results: Solid-state characterization studies revealed that ketoprofen was present in a solid solution state in the hot-melt extruded polymer matrix. The desired responses of the prepared optimized KTP pellets obtained by considering the designed space showed 1.20% drug release in 2 h, 3.73% in the first 5 h of the lag period with the help of Eudragit^®^ S 100 coating, and 93.96% in extended release up to 24 h in the colonic region. Conclusions: Hence, developing Eudragit-coated hot-melt extruded pellets could be a significant method for achieving the colon-specific release of ketoprofen.

## 1. Introduction

Colon-specific drug delivery has grabbed interest recently because of its potential benefits in treating various diseases, including colon cancer, Crohn’s disease, and ulcerative colitis [1,2,3]. Drugs that are targeted to the colon have the potential to improve drug absorption, decrease systemic drug exposure and its associated toxicity, and enhance disease-specific treatment [4,5]. Colon-specific drug delivery is aimed at preventing unintended upper-GIT drug release by selectively releasing medicament in the colon [6,7,8]. In recent years, numerous approaches that have been explored to achieve colon-specific drug delivery include pH-responsive, time-controlled, enzyme-activated, magnetically controlled, and receptor-targeted (designed explicitly for overexpressed receptors at the site of the disease) systems [9,10].

The colon pH is substantially higher than that of the upper GIT. Colon-specific drug delivery systems can be developed using pH-responsive polymers like methacrylate copolymers [11]. Eudragit^®^ S 100 and L 100 polymers are extensively utilized to attain colon-specific drug delivery due to their ability to release drugs in a pH-dependent manner [12,13]. However, the pH-dependent delivery systems show inconsistent results due to variations in pH, volume of fluids, gastrointestinal transit times, and motility among individuals. Furthermore, disease condition and food intake will also affect these factors, diminishing the effectiveness of drug-release systems that rely on pH, often resulting in drug release at undesired sites [14]. To conquer the above problems and to minimize early upper-GIT drug release, a system established on a combination approach using polysaccharides and pH-dependent polymers can be developed [15].

Polysaccharides can be considered significant options for achieving colon-specific drug delivery [16] because of their high gelation strength and degradation in the presence of colonic microbial flora. Pectin can be a good option as an extended-release matrix for colon targeting due to its gelling capacity and enzyme-triggered response in the colon [17,18]. Hence, combining pectin (enzyme-triggered) and Eudragit^®^ S 100 (pH-dependent polymer) works synergistically to provide a site-specific release for colonic delivery. Colonic microflora release enzymes that can break down the enzyme-sensitive component, if failed in the polymer’s pH dissolution threshold. This extra safety mechanism circumvents the problems with regular pH-dependent systems [19].

Ketoprofen (KTP), a non-steroidal anti-inflammatory drug, is often utilized in rheumatoid arthritis treatment as well as for postoperative and trauma pain due to its analgesic and antipyretic characteristics [20]. Gastric ulceration and bleeding are the most frequent adverse reactions to KTP [21]. KTP is an excellent option for colon delivery to reduce its toxicity in the upper GIT. Furthermore, KTP’s thermal stability is one of the reasons for its selection as a model drug in this study.

In our previous study, ketoprofen pellets were prepared to target the colon using pectin as an enzyme-triggered carrier and HPMC as a time-dependent polymer to prevent upper-GIT drug release [17]. A high early drug release (~13%) with a slow release (~60%) over 24 h was observed. The formulations were prepared traditionally by considering one factor at a time using two polymer systems (HPMC and Pectin). With the previous experience, an effort was made to lower the premature upper-GIT drug release and increase the extended drug release in the colon by employing a pH-responsive Eudragit^®^ S 100 and time-dependent polymer (HPMC HME 4M) matrix combined with enzyme-triggered polysaccharide (Pectin LM). The current work is designed to optimize the concentration of Eudragit^®^ S 100, HPMC HME 4M, and Pectin LM polymer matrix for targeting KTP to the colon using a QbD approach.

Quality by Design (QbD) is a methodological approach to manufacturing pharmaceutical products, emphasizing predefined objectives. This approach facilitates the understanding of both the process and the product while optimizing the utilization of resources such as time, money, and effort [22,23]. The Design of Experiments (DoE) is a structured and systematic approach employed to examine the impact of factors on desired responses within predefined ranges [22]. Meanwhile, Response Surface Methodology (RSM) is a statistical technique that addresses challenges in optimizing drug formulation and enables the prediction of connections between factors and responses. It proves highly efficacious in optimizing independent variables and forecasting responses based on pertinent experimental data derived from a regression analysis model [23].

This investigation involved preparing and optimizing colon-targeting pellets using QbD principles by employing a Box–Behnken Design (BBD). The optimized formulation underwent a comprehensive evaluation to assess the premature upper-GIT release and the extended drug release in the colon. Different concentrations of Pectin LM (A), HPMC HME 4M (B), and Eudragit^®^ S 100 (C) served as the independent variables. The investigation focused on assessing the various responses of KTP pellets, encompassing the % drug release in 2 h (Q2; Y1), % drug release in 5 h (Q5; Y2), and % drug release in 24 h (Q24; Y3).

The present study was intended to check the potential of an enzyme-triggered system in the presence of a pH-responsive system (Eudragit^®^ S 100) to achieve the colon-specific drug delivery of KTP using HPMC and pectin by hot-melt extrusion (HME). HME is a continuous manufacturing process that could facilitate the development of colon-specific drug delivery systems. The aim was to minimize the upper-GIT drug release and enhance the extended drug release in the colon by using Response Surface Methodology (RSM). The study delves into examining the impact of different polymer concentrations on product performance, employing a BBD. Furthermore, successful implementation of the above strategy may demonstrate the feasibility of shielding the medicament from upper-GIT degradation and safeguarding the upper GIT from drug-induced ulcers and bleeding.

## 2. Materials and Methods

### 2.1. Materials

KTP was procured through PCCA located in Houston, TX, USA. HPMC HME 4M and Pectin LM were bought from Colorcon Inc., West Point, PA, USA, and CP Kelco—Atlanta, GA, USA respectively. Eudragit^®^ S 100 was obtained from Evonik, Essen, Germany. Pectinex^®^-Ultra-SP-L was bought from Modernist Pantry LLC—Eliot, ME, USA.

### 2.2. HPLC Analysis for KTP

KTP concentrations in in vitro samples were determined with the aid of a Waters HPLC system using a C18 reverse phase column (Phenomenex Luna^®^) and UV detector (Waters Corp., Milford, MA, USA). The mobile phase is composed of water, acetonitrile, and glacial acetic acid (90:110:1). The following parameters were used to run the analysis: flow rate—1.2 mL/min, wavelength—256 nm, and injection volume—20 µL [17].

### 2.3. Design of Experiment and Development of Design Space

A Box–Behnken Design (BBD) was constructed to optimize three formulation variables and assess their impact on responses using Design-Expert^®^ software (version 13). Table 1 presents the factors, codes, and actual values used to construct the BBD. The independent factors, namely Pectin LM (A), HPMC HME 4M (B), and Eudragit^®^ S 100 (C), were examined at three levels: −1, 0, and +1. A total of 17 experiments were conducted, and the analysis focused on three dependent variables: % drug release in 2 h (Q2; Y1), % drug release in 5 h (Q5; Y2), and % drug release in 24 h (Q24; Y3). The software determined *f*-values, *p*-values, and estimated coefficients. The developed model, a third-order polynomial equation, is described as:

Y = β0 + β1 A + β2 B + β3 C + β11 A^2^ + β22 B^2^ + β22 C^2^ + β123 ABC (1)

“Y” denotes the predicted responses for the percentage drug release in 2 h, 5 h, and 24 h. A, B, and C represent the factors; the coefficients include intercept (β0), linear coefficients (β1 and β2), square coefficients (β11, β22, and β33), and interaction coefficient (β123).

Additionally, the model validation process was thorough, involving a detailed comparison between predicted and observed values. This approach helped establish a flexible design space for each response criterion, taking desirability into account. Consequently, the optimized batch was meticulously manufactured using this design space with the optimal values.

### 2.4. Physical Mixtures for HME

Table 2 displays the various compositions utilized in the hot-melt extrusion process. Following a geometric dilution procedure, the required amounts of formulation excipients were mixed with KTP. They were blended for 15–20 min using a V-blender (MaxiBlend™, GlobePharma, Monmouth Junction, NJ, USA) at optimum speed to ensure homogeneous drug content throughout the produced pellets. The samples were subjected to a sieving process using mesh #45 to ensure no aggregates were present in the mixture and evaluated for blend uniformity. An accurately weighed amount of 0.5 g was collected from three different locations in each physical mixture and dissolved in 100 mL of mobile phase. The filtrate was quantified for KTP using the HPLC method described above to check blend uniformity.

### 2.5. Pellets Preparation Using HME

Process 11™, a twin-screw corotating extruder of 11 mm size (ThermoFischer Scientific—Austin, TX, USA) combined with a Varicut pelletizer (ThermoFischer Scientific GmbH, Dreieich, Germany) was used to prepare the KTP pellets. Mixtures of samples containing the required quantity of KTP (50 mg), HPMC HME 4M (100 mg, 125 mg, and 150 mg), and Pectin LM (50 mg, 75 mg, and 100 mg) were effectively expelled through extrusion at a screw speed of 50 rpm, a 5-set feed rate (1–2 g/min), and a temperature of 115 °C in all barrel zones. The extruder barrel and the die were heated until they reached a sufficient temperature. This was then allowed to thermally stabilize for 15 min before commencing the processing. The heated extrudate was subjected to elongation using a conveyor belt for every processing method, and an initial 5.0 g of hot-melt extrudate was removed. Then, the filaments (extrudates) underwent a cooling and hardening process before being fed into the Varicut pelletizer. The pellets were acquired during consistent extrusion, and the speed at which the pellets were formed was aligned with the same rate of extrusion. Pellets weighing around 15 g were collected and kept in airtight plastic bags at 20–25 °C for each formulation. Subsequently, pellets equivalent to 50 mg of KTP were fed manually into the 00-sized shells of hard gelatin capsules.

### 2.6. Preparation of Eudragit^®^ S 100 Coated Pellets

The coating solution was prepared by dissolving different concentrations of 5%, 10%, and 15% *w*/*v* of Eudragit^®^ S 100 in acetone by adding a suitable amount of plasticizer (triethyl citrate) and anti-sticking agent (talc). Then, the core pellets were coated using the Freund Vector LDCS 5 laboratory coating system until the cores reached 20% *w*/*w* weight gain. The coating system was optimized and operated at 10 rpm pan speed, 5 rpm pump speed, 60 °C inlet air temperature, and 12 PSI nozzle pressure to ensure the uniform coating of the pellets.

### 2.7. Drug Content (Assay)

A precisely measured quantity (1.0 g) of the developed pellets was pulverized in a porcelain mortar to obtain a finely powdered material. An accurately weighed quantity of powder material equivalent to 50 mg of KTP was taken in a 100 mL volumetric flask. The mobile phase was added to the flask and kept for 10 min sonication using the Branson Ultrasonic sonicator. After that, the samples were centrifuged for 10 min at 12,000 rpm & 25 °C and passed through a 0.45 μm Nylon membrane filter. Following that, the filtrate underwent a 10-fold dilution with the mobile phase and was subjected to analysis for drug content with the HPLC method given in Section 2.2.

### 2.8. In Vitro Drug Release Studies

Dissolution testing was conducted in three stages utilizing a Hanson SR8-plus TM dissolution apparatus (Hanson Research, Chatsworth, CA, USA) in three distinct media [24]. The speed of the basket was set to 100 rpm, while the dissolution media comprised a 900 mL volume. The dissolution medium was subjected to thermal equilibration at 37 ± 0.5 °C. Then, dissolution was studied in 0.1 N HCl (first 2 h) followed by 6.8-pH phosphate buffer for the next 3 h, and finally in the 7.4-pH phosphate buffer with 3 mL of Pectinex^®^ UltraSP-L enzyme (simulated colonic fluid) for 24 h [17]. About 1.5 mL of samples were taken at consistent time points and substituted with the same amount of blank solution [25]. The analysis of the samples was performed using the previously described HPLC method for dissolved drug content. Notably, the dissolution testing for the lead formulation was conducted again without the use of an enzyme. The study was conducted in triplicate (*n* = 3) and the mean of the cumulative percentage drug release was calculated.

### 2.9. Thermal Studies

The thermal behavior of the drug was examined using a differential scanning calorimeter, i.e., a Discovery instrument (DSC 25, TA Instruments, New Castle, DE, USA) [26]. The fine powder from grinding the pellets was used to prepare 5–10 mg samples, which were subsequently placed in hermetically sealed aluminum pans. In an ultra-purified nitrogen atmosphere with a 50 mL/min purge flow, the samples were heated from 25 to 200 °C at 10 °C per minute. DSC thermograms were plotted using Trios software (TA Instrument-DE). Furthermore, the crystalline nature of the drug was proved by investigating powder X-ray diffraction (PXRD). This analysis was done for both pure drug and formulation, as discussed in our previous article, over a 2–50° scanning range [17].

### 2.10. Accelerated Stability Studies

Formulation capsules were packed in glass vials and kept at 40 ± 2 °C and 75 ± 5% RH (accelerated circumstances for zone I) for six months and studied for the in vitro dissolution studies along with drug content estimation.

## 3. Results and Discussion

### 3.1. HPLC Analysis for KTP

The concentrations from 5–100 µg/mL were found to be linear, as demonstrated by a six-point calibration curve with an R^2^ of 0.9991. The method’s sensitivity was determined to be 0.2 µg/mL, and its quantitation limit to be 0.7 µg/mL.

### 3.2. Design of Experiment and Development of Design Space

Colon-specific drug delivery systems of KTP were developed by employing a pH-responsive Eudragit^®^ S 100 and time-dependent polymer (HPMC HME 4M) matrix combined with enzyme-triggered polysaccharide (Pectin LM). In the preparation of colon-specific release pellets, the independent factors (variables), namely Pectin LM, HPMC HME 4M, and Eudragit^®^ S 100 (Table 1), were chosen. In the present investigation, a BBD was implemented to optimize these variables. Table 2 shows the levels of independent factors, the number of experiments conducted, and their corresponding responses. The Analysis of Variance (ANOVA) was performed employing Design Expert^®^ (version 13) to assess the influence of variables on the responses. A thorough analysis of the model sum of squares and lack of fit was carried out, considering 2-factor interaction (2FI), linear, quadratic, and cubic models [27]. According to the guidelines provided by the Design Expert^®^, a regression model is deemed significant under the following conditions: the model *f*-value should be significantly greater than 1, with a *p*-value of <0.05; lack of fit *p*-value exceeds 0.05, indicating non-significance; predicted R^2^ and adjusted R^2^ difference is less than 0.2; the adequate precision is greater than 4 [28,29].

### 3.3. HME and Pelletization

HME process parameters (specifically feed rate & screw speed) were adjusted during preliminary trials, and the same parameters were used for performing all experiments. Based on preliminary trials, the temperature in all the barrel zones was optimized to be 115 °C, which reduced the degradation. All formulations were extruded using a screw configuration that adhered to standard specifications, featuring three mixing zones. The resulting strands, after passing through the die cavity, were found to be constant in size and shape (around 4 mm and circular). The procedure was unsuccessful when KTP and pectin physical blends were subjected to the HME process at a 115 °C temperature, and the extrudate turned dark brown to black when the processing temperature was raised to 130 °C, due to the degradation of pectin [30]. Adding HPMC to the pectin enhanced the processing of pectin polymer at low temperatures, while simultaneously augmenting the quality of extrudate/filament. The drug load of the extrudates was similar to the theoretical values, and the HPLC chromatograms did not exhibit any new or unusual peaks when assessed with a physical mixture, demonstrating the stability of the drug at HME processing conditions. Table 3 displays the data for blend uniformity of the physical mixtures and the assay of prepared pellets for all the formulations.

### 3.4. In Vitro Drug Release Studies

**Figure 1:**
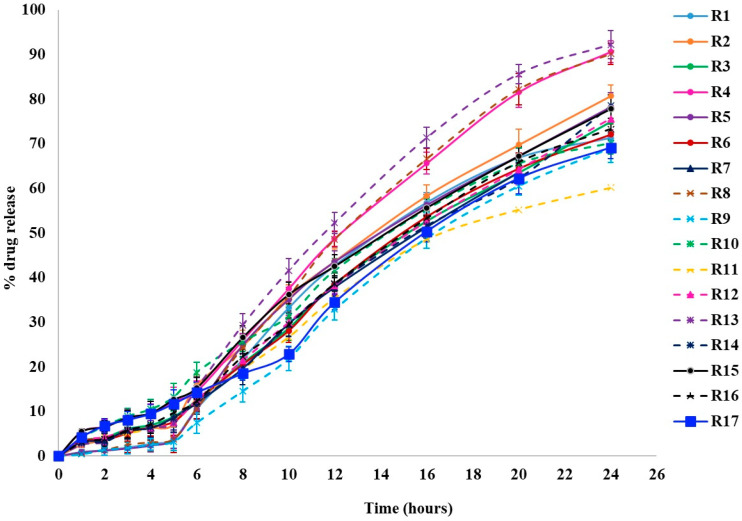
Drug release profiles of KTP colon-specific release pellets.

Dissolution studies were conducted to examine the release behavior of KTP from pellets, using capsules filled with either uncoated or coated pellets in three different pH media to simulate the stomach, small intestine, and colonic regions of the GIT. The results are represented in Figure 1. The Q values at different time points are mentioned in Table 2.

#### 3.4.1. Cumulative Percentage Drug Release in 2 h (Q2)

The first step in drug release studies for colonic systems is to observe the drug release in 0.1 N HCl for the first 2 h. In the previous study, the premature drug release rate was slightly higher. This may be attributed to the absence of a pH-responsive polymer. In this study, Eudragit^®^ S 100 (5%, 10%, and 15%) was utilized to reduce premature drug release. An increase in the concentration of Eudragit^®^ S 100 decreases the % of drug release. All formulations coated with Eudragit^®^ S 100 were able to reduce the drug release from 6.83% to 1.25% in the first 2 h. The negligible drug release in the upper GIT prevents the loss of KTP and reduces its side effects. Among all formulations, R1, R5, R8, and R9 have higher levels of Eudragit^®^ S 100 (15%) and showed a very low % drug release (1.25% to 1.42%). The optimized formulation showed only 1.19% drug release in 0.1 N HCl over 2 h. HPMC HME 4M and Pectin LM did not significantly impact drug release in the first 2 h. These results were evident by the following results observed from DOE studies.

The range of the % drug release was from 1.25% to 6.84%. According to statistical regression analysis, a reduced linear model is the optimal choice for examining the impact of variables on the % drug release. The regression model exhibits a notable *f*-value of 516.95, implying the model is statistically significant. The *p*-value of less than 0.0001 suggests the significance of the model terms. In this instance, the C (Eudragit^®^ S 100) model term is statistically significant. Furthermore, a lack of fit (*p*-value of 0.4130) is non-significant (Table 4). For the model’s overall fit, lack of fit should be statistically not significant.

**Figure 2:**
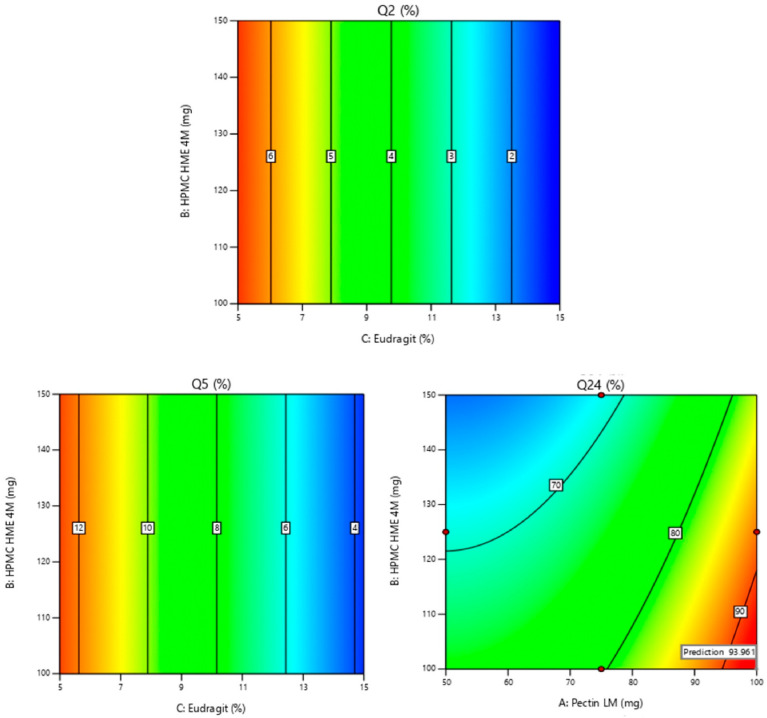
2-D contour plots of Y1: % drug release in 2 h (Q2); Y2: % drug release in 5 h (Q5); Y3: % drug release in 24 h (Q24). Note: Contour plots display the predicted outcomes (like Q2, Q5, or Q24), and the red dots indicate the real values obtained during experiments at those specific conditions. They are used to show how well the experimental data aligns with the model’s predictions. The values displayed on the Contour plots indicate the percentage of drug release in response to factors shown on the axis. Q2 & Q5: As the concentration of Eudragit increases, the % drug release decreases. Q24: As the concentration of Pectin increases the % drug release increases.

**Figure 3:**
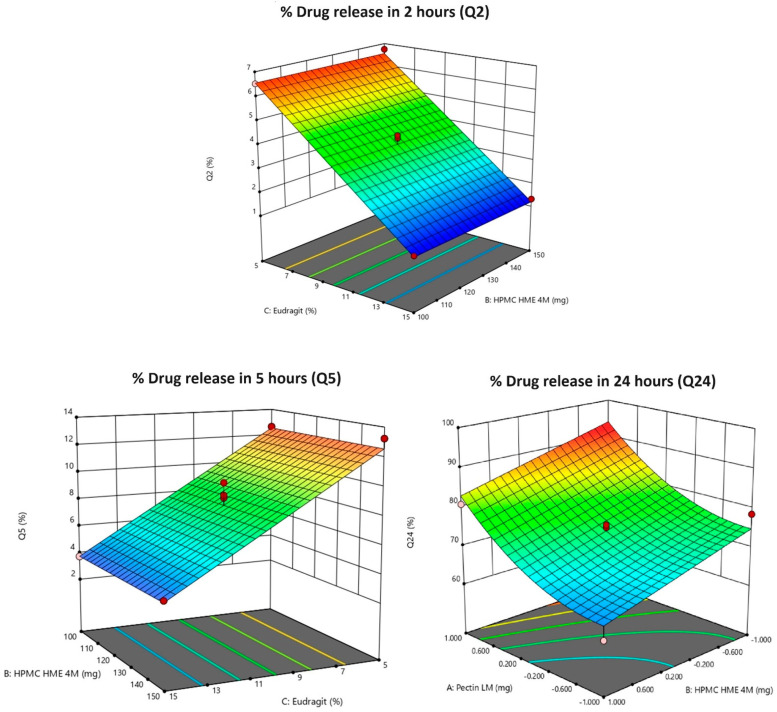
3D response surface plots of Y1: % drug release in 2 h (Q2); Y2: % drug release in 5 h (Q5); Y3: % drug release in 24 h (Q24). Note: 3D response surface plots display the predicted outcomes (like Q2, Q5, or Q24), and the red dots indicate the real values obtained during experiments at those specific conditions. They are used to show how well the experimental data aligns with the model’s predictions. Pink dots indicate the corner points for the responses (Q2, Q5, and Q24).

Results from Table 2 and Table 3 show Eudragit^®^ S 100 (C) has a significant impact on the Y1 (the *p*-value is less than 0.0001), characterized by a negative coefficient (−2.67). A rise in the quantity of Eudragit^®^ S 100 (C) results in a significant decrease in Y1. The consistency between the adjusted R^2^ (0.9699) and predicted R^2^ (0.9668), with a difference of less than 0.2, is considered reasonable. The observed adequate precision also exceeds the desirable threshold of 4, with a value of 46.873. The 2D and 3D surface response plots, illustrated in Figure 2 and Figure 3, respectively, visually represent these effects. The relative impact of the factors can be determined by comparing the coefficients in the coded equation. The equation used to analyze Y1 in terms of coded factors is represented by the coded equation [31].

Y1 = 3.87 − 2.67 C(2)

where Y1: Predicted responses for % drug release in 2 h (Q2), C: Concentration of Eudragit^®^ S 100.

**Figure 4:**
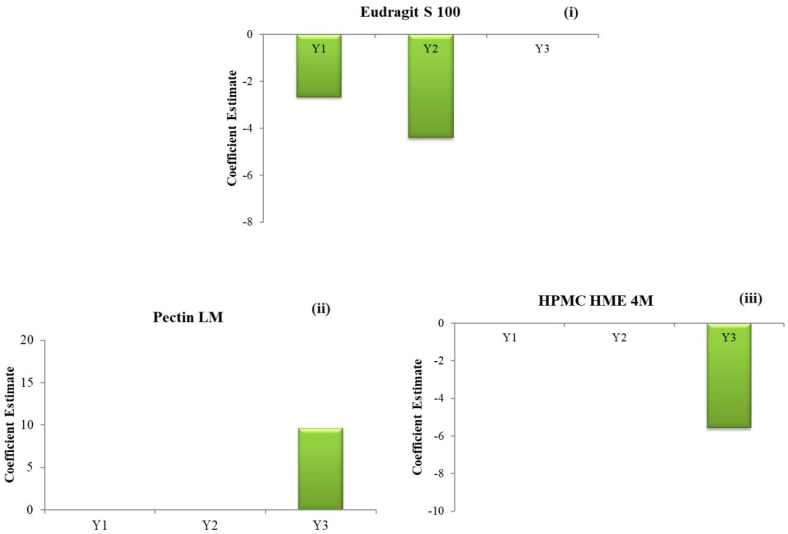
Coefficient estimates of the factors (**i**) % drug release in 2 h (Q2); (**ii**) % drug release in 5 h (Q5); (**iii**) % drug release in 24 h (Q24).

The outcomes demonstrate a significant (negative) influence of the concentration of Eudragit^®^ S 100 (C) on the drug release, as depicted in Figure 4(i)–(iii). Conversely, the concentrations of HPMC HME 4M and Pectin LM exhibit a negligible impact on the Q2.

#### 3.4.2. Cumulative Percentage Drug Release in 5 h (Q5)

The second step is releasing the drug into a 6.8 pH phosphate buffer for 5 h. As the pH of the dissolution medium increases, the premature drug increases slightly compared to the % drug release in 0.1 N HCl. This is due to the dissolution of the Eudragit^®^ S 100 coat in the pH 6.8 phosphate buffer. The % drug release is from 3.25% to 13.24% in 5 h. A higher level of Eudragit^®^ S 100 (15%) still shows very low premature drug release (3.35% to 3.86%) from R1, R5, R8, and R9 formulations. HPMC HME 4M and Pectin LM will not have a significant impact on drug release in the first 2 h and 5 h. These results were evident by the following results observed from DOE studies.

The range of the % drug release was from 3.25% to 13.24%. According to statistical regression analysis, the reduced linear model is the optimal choice for examining the impact of variables on the % drug release. The regression model exhibits a notable *f*-value of 321.89, implying the model is statistically significant. The *p*-value of less than 0.0001 suggests the significance of the model terms. In this instance, the C (Eudragit^®^ S 100) model term is statistically significant. Furthermore, a lack of fit (*p*-value of 0.8813) is non-significant (Table 3). For the model’s overall fit, lack of fit should be statistically not significant.

Results from Table 2 and Table 3 show Eudragit^®^ S 100 (C) has a significant impact on the Y2 (the *p*-value is less than 0.0001), characterized by a negative coefficient (−4.41). A rise in the quantity of Eudragit^®^ S 100 (C) results in a significant decrease in Y2. The consistency between the adjusted R^2^ (0.9525) and predicted R^2^ (0.9463), with a difference of less than 0.2, is considered reasonable. The observed adequate precision also exceeds the desirable threshold 4, measuring 36.9869. The 2D and 3D surface response plots, illustrated in Figure 2 and Figure 3, respectively, visually represent these effects. The relative impact of the factors can be identified from the coded equation by comparing the factor coefficients. The coded equation used to analyze Y2 in terms of coded factors is represented by the equation [32]

Y2 = 8.14 − 4.41 C(3)

where, Y2: Predicted responses for % drug release in 5 h (Q5), C: Concentration of Eudragit^®^ S 100.

The outcomes demonstrate a significant (negative) influence of the Eudragit^®^ S 100 concentration (C) on the % drug release, as depicted in Figure 4(i)–(iii). Conversely, the concentrations of HPMC HME 4M and Pectin LM have a negligible impact on the Q5.

#### 3.4.3. Cumulative Percentage Drug Release in 24 h (Q24)

The third step is the release of the drug in a 7.4-pH phosphate buffer with 3 mL of Pectinex^®^ UltraSP-L (Modernist Pantry LLC, Elliot, ME, USA) enzyme (simulated colonic fluid) for 24 h. In the previous work, the % of the drug release was slower in 24 h and this may be due to the lower level of an enzyme-triggered polysaccharide (Pectin). In the current study, Pectin LM of 50 mg, 75 mg, and 100 mg was used along with a time-dependent polymer, i.e., HPMC in amounts of 100 mg, 125 mg, and 150 mg, to increase the % drug release in 24 h.

The % drug release was found to be 60.17 to 92.26% over 24 h. The increase in the concentration of HPMC HME 4M decreases the % of drug release, which may be due to the inhibition of diffusion of the drug. Conversely, an increase in the concentration of Pectin LM increases the release of the drug in the Pectinex^®^ UltraSP-L enzyme. Pectinex^®^ UltraSP-L enzyme breaks down the structure of Pectin, and the drug will diffuse out of the matrix. The higher the amount of Pectin LM in the pellet, the higher amount of drug will diffuse from the matrix due to enzymatic degradation in the colon. That is why a larger amount of the drug was released at higher levels of Pectin LM. If only HPMC HME 4M is used in the matrix, it will inhibit the release of the drug. If only Pectin LM is used in the matrix, the extended release of the drug cannot be maintained. In order to maintain the extended release of the drug in the colon, pellets were prepared with the addition of a time-dependent polymer (HPMC HME 4M) and enzyme-triggered polysaccharide (Pectin LM). Eudragit^®^ S 100 does not have a significant impact on drug release in the first 24 h. These results were evident by the following results observed from DOE studies.

The range of the % drug release was from 60.17% to 92.26%. According to statistical regression analysis, a reduced quadratic model is the optimal choice for examining the impact of variables on the % drug release. The regression model exhibits a notable *f*-value of 71.49, implying the model is statistically significant. The *p*-value of less than 0.0001 suggests the significance of the model terms. In this instance, A, B, and A^2^ model terms are statistically significant. Furthermore, a lack of fit (*p*-value of 0.1403) is non-significant (Table 3). For the model’s overall fit, lack of fit should be statistically not significant.

Results from Table 2 and Table 3 show ‘A’ has a significant impact on the Y3 (the *p*-value is less than 0.0001), characterized by a positive coefficient of 9.61, and ‘B’ has a significant impact on the Y3 (the *p*-value is less than 0.0001), characterized by a negative coefficient of −5.57. A rise in the quantity of Pectin LM (A) results in a significant increase in Y3. A rise in the quantity of HPMA HME 4M (B) results in a significant decrease in Y3. The consistency between the adjusted R^2^ (0.9297) and predicted R^2^ (0.8800), with a difference of less than 0.2, is considered reasonable. The observed adequate precision also exceeds the desirable threshold of 4, measuring 27.8451. The 2D and 3D surface response plots, illustrated in Figure 2 and Figure 3, respectively, visually represent these effects. The relative impact of the factors can be determined from the coded equation by comparing the factor coefficients. The coded equation used to analyze Y2 in terms of coded factors is expressed by the equation [32]

Y3 = 74.06 + 9.61 A − 5.57 B + 4.48 A^2^ (4)

where Y3: Predicted responses for % drug release in 5 h (Q24), A: Concentration of Pectin LM, B: Concentration of HPMC HME 4M.

The outcomes demonstrate a significant positive influence from the concentration of Pectin LM (A) and a significant negative influence from the concentration of HPMC HME 4M (B) on the % drug release, as depicted in Figure 4(i)–(iii). Conversely, the Eudragit^®^ S 100 concentration exhibits a negligible impact on the Q24.

### 3.5. Regression Analysis and Verification of Model Adequacy

The optimization data analysis involved fitting the experimental data to a suitable mathematical model for the evaluated responses. The model was constructed by selecting model parameters, and multiple linear regression analysis (Table 4). ANOVA, correlation coefficient, and lack of fit were considered to evaluate the model’s adequacy. Furthermore, the relationship between factors and responses was analyzed through response surface mapping using both 2D and 3D plots.

**Figure 5:**
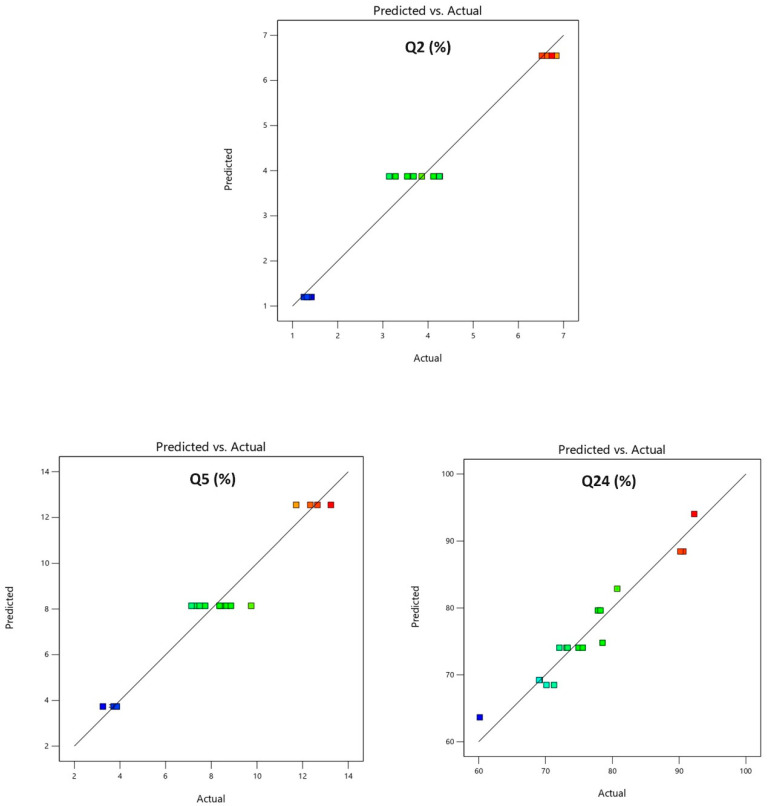
Predicted vs. actual plots Y1: % drug release in 2 h (Q2); Y2: % drug release in 5 h (Q5); Y3: % drug release in 24 h (Q24).

The model’s accuracy was subsequently evaluated with predictability plots. Predicted values were compared to experimental values to assess the regression model’s adequacy for Y1, Y2, and Y3. The comparison results are presented in Table 5 and Figure 5. The predicted values closely matched the actual values, suggesting a realistic fit for the regression model and good predictability.

### 3.6. Multiple Responses Optimization Study

The optimized amounts of Eudragit^®^ S 100, HPMC HME 4M, and enzyme-triggered Pectin LM that are required to prevent premature release in the upper GIT and increase colonic drug release can be obtained from a multiple response optimization approach. A numerical and graphical optimization approach was used to optimize various responses by considering the desirability function. The formulation’s optimum ranges of independent variables (studied factors) were estimated using desirability as a mathematical method [32]. The desirability function ranges from 0 to 1. A value of 0 indicates a completely undesirable response, while 1 signifies the most desirable response [33].

**Figure 6:**
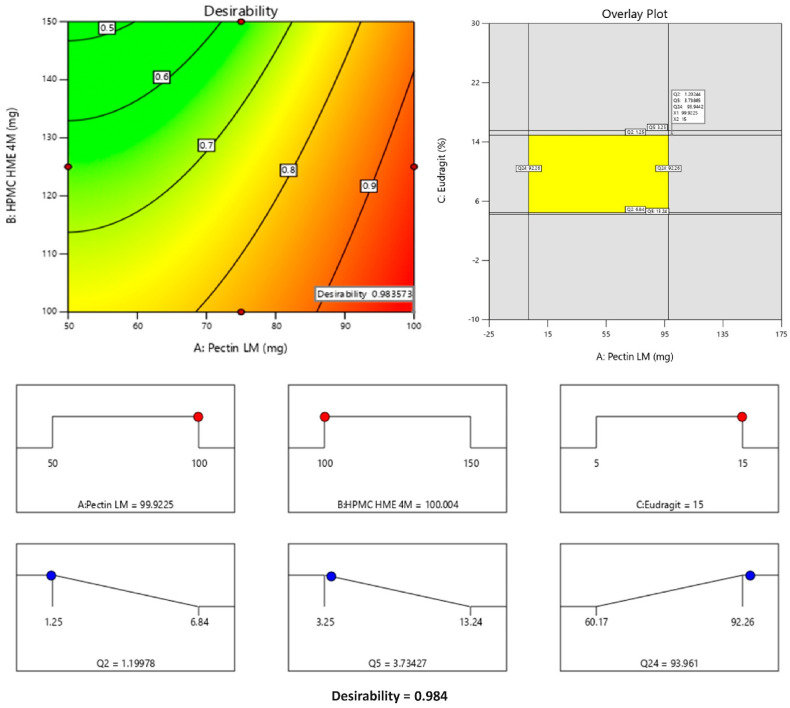
Levels of factors, design space, and desirability for the optimized formulation. Note: The Counter plot displays the predicted outcomes (like Q2, Q5, or Q24), and the red dots indicate the real values obtained during experiments at those specific conditions. They are used to show how well the experimental data aligns with the model’s predictions. The yellow area typically represents the design space or optimal region where all the criteria or constraints are satisfied. This means that within this region, the responses (in this case, Q2, Q5, and Q24) fall within the desired or acceptable ranges. The red dots represent the maximum values of the input factors (A: Pectin LM, B: HPMC HME 4M, and C: Eudragit), indicating the upper levels of these variables in the study. The blue dots correspond to the predicted responses (Q2, Q5, Q24) based on the chosen conditions, showing how each response changes within the range of input values. The desirability score of 0.984 reflects the optimal conditions predicted by the model, maximizing the response variables while staying within the specified constraints. The values are an indication of desirability in response to the factors presented on the axis. As the concentration of Pectin increases the desirability value increases. In the overlay plot, the values represent Q2, Q5, and Q24 at optimized concentrations of factors like Eudragit, HPMC, and Pectin.

The formula comprising 100 mg of Pectin LM, 100 mg of HPMC HME 4M, and 15% Eudragit^®^ S 100 was selected as the optimized formulation, achieving a desirability value of 0.984 as depicted in Figure 6. This optimized formula demonstrated lower premature drug release in the upper GIT (1.20% in 2 h and 3.73% in 5 h) and increased drug release in the colon (93.96% in 24 h). The optimized formula then advanced to subsequent characterization studies for further evaluation.

### 3.7. Effect of Pectinex^®^ Enzyme on Drug Release

**Figure 7:**
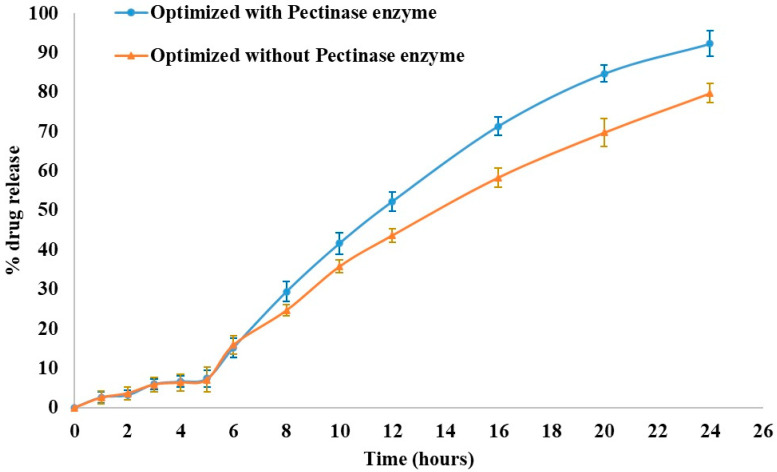
Effect of Pectinex^®^ enzyme on the dissolution profile of optimized formulation.

To examine the impact of enzymatic degradation, a dissolution study was conducted with and in the absence of Pectinex^®^ Ultra SP-L enzyme in the simulated colonic fluid (Figure 7). There was no significant difference between the two profiles (with and without the degrading enzyme) regarding the percentage of KTP released in the first 5 h. When the matrix-degrading enzyme was included in the pH 7.4 phosphate buffer, the dissolution rate was increased to 92.26% after 24 h from 79.73% (without enzyme). Many previous studies [17,34] corroborated these findings.

### 3.8. Uniformity of Blend and Drug Content

Blend uniformity determines the effective mixing of drugs and excipients used in the formulation. Poor blend uniformity leads to non-uniform distribution of the drugs in the blend, which can lead to poor/improper results in the final dosage form. The drug content (Assay) in pellets was also measured. Table 5 shows that the drug content and the blend uniformity of all the formulations ranged from 90 to 110%, confirming the employment of a highly effective PM preparation and extrusion process.

### 3.9. Thermal Studies

**Figure 8:**
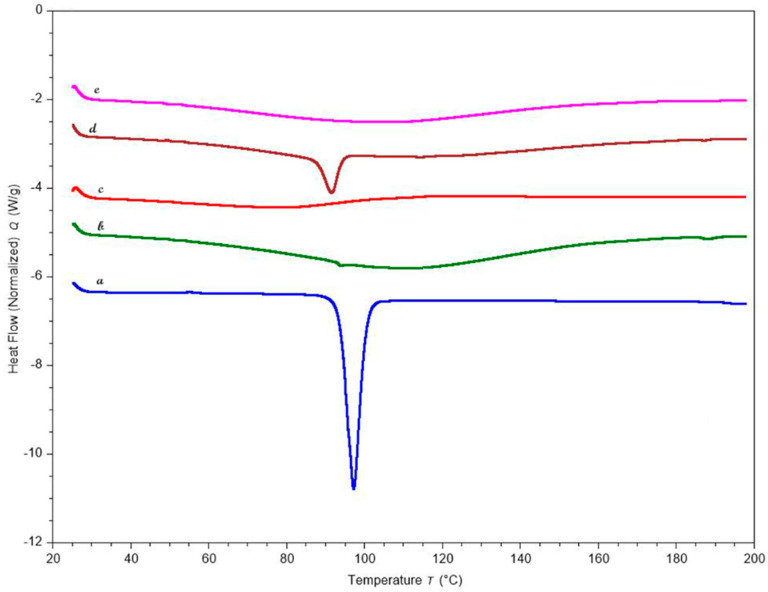
DSC curves of (**a**) KTP (**b**) Pectin LM (**c**) HPMC HME 4M (**d**) Optimized formulation physical blend (**e**) Optimized formulation pellets.

DSC is an effective thermal method for evaluating and distinguishing thermal transitions in drug and polymeric materials. Figure 8 displays the thermograms of KTP, polymers, and the optimized formulation. The endothermic melting peak of the drug appeared in the DSC curves at about 94 °C. After the pellets were formed, the DSC curves showed no trace of the KTP peak, indicating that the crystalline drug had been completely solubilized in the chosen polymeric carriers and had changed to an amorphous state.

**Figure 9:**
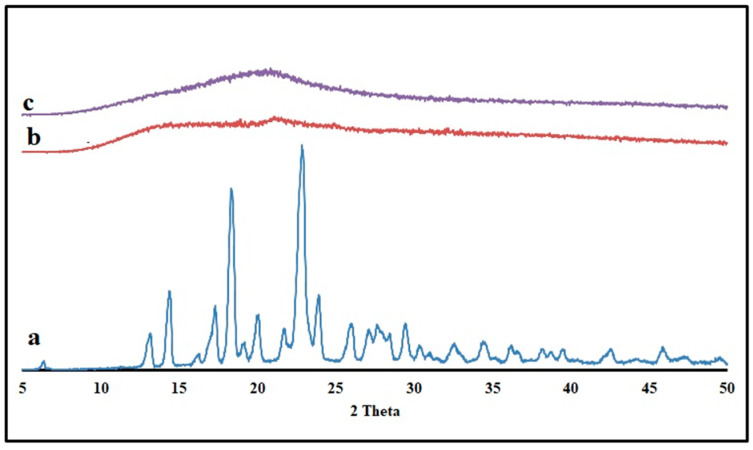
Powder X-ray diffractograms of (**a**) KTP (**b**) Pectin LM (**c**) Optimized formulation pellets.

The drug’s solid-state transition was measured for KTP and optimized formulation using the powder X-ray diffraction (PXRD) method (Figure 9). Pure KTP is crystalline, as evidenced by two prominent peaks in the PXRD pattern at two angles of 18.38° and 22.84° [32]. Crystalline KTP may have been converted to an amorphous state, as the pellet’s PXRD patterns showed no trace of the drug’s characteristic peaks. The high mixing shear and temperature during the HME process were found to be responsible for this thermal transition from the crystalline to amorphous state in polymers. These results proved the solid-solution form of KTP in the extruded pellets.

### 3.10. Accelerated Stability Studies

**Figure 10:**
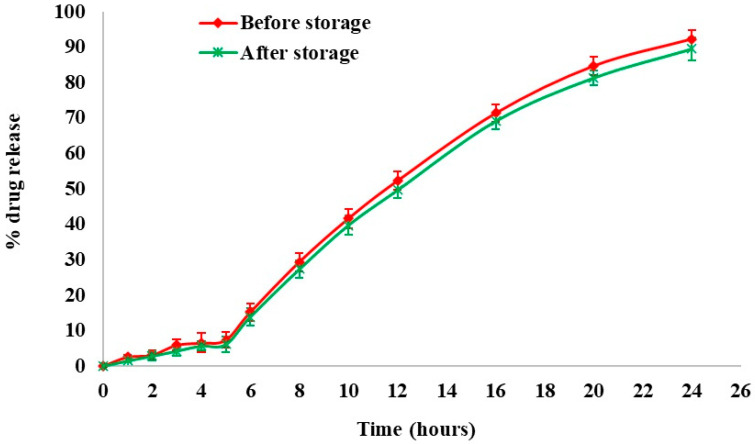
Stability studies—dissolution profile of optimized formulation before and after storage.

At regular intervals, physicochemical stability was assessed by dissolution studies and drug content measurement. Figure 10 depicts the effect of an accelerated storage environment on drug release behavior. From the results, the optimized formulation was found to be stable under accelerated stability conditions for 6 months. The similarity index value between the dissolution profiles of the optimized formulation before and after storage was determined to be 72.64.

## 4. Conclusions

An attempt was made to develop Eudragit^®^ S 100-coated pectin–HPMC-based pellets to attain the colonic release of KTP, not only to minimize its side effects in the upper GIT by reducing drug release, but also to increase its therapeutic efficiency by maximizing drug release in the colon. Box–Behnken design coupled with the Response surface method was used to design the experiments. Initially, the drug-loaded pectin–HPMC-based pellets were successfully prepared using HME coupled with a die-surface cutting pelletizer, and then Eudragit^®^ S 100 coating was applied to reduce this initial drug release in the upper GIT over 2 h (1.20%) followed by over 5 h (3.73%), i.e., in lag time. The pellets showed an increased drug release (93.96%) in the colon for 24 h, which was observed with an increased amount of Pectin LM combined with a low level of HPMC HME 4M. Hence, these results proved that the combination of a pH-dependent approach with an enzyme-triggered approach is a significant way to obtain the colon-specific delivery of KTP without a significant drug loss in the stomach and small intestine.

## Figures and Tables

**Table 1:** Independent variables (Factors) and levels encompassed by the Box–Behnken design.

Levels	Factors (Variables)		
	HPMC HME 4M (mg)	Pectin LM (mg)	Eudragit^®^ S 100 (%)
−1	100	50	5
0	125	75	10
+1	150	100	15

**Table 2:** Results of Box–Behnken Design and the Responses (Dependent Variables) Obtained for Experimental Run.

Standard Order	Experimental Run	Factor A: Pectin LM (mg)	Factor B: HPMC HME 4M (mg)	Factor C: Eudragit^®^ S 100 (%)	Response Y1	Response Y2	Response Y3
					Q2 (%)	Q5 (%)	Q24 (%)
12	R1	0	1	1	1.32	3.86	71.28
4	R2	1	1	0	3.65	7.13	80.73
15	R3	0	0	0	4.12	8.86	74.94
6	R4	1	0	−1	6.63	12.34	90.63
11	R5	0	−1	1	1.25	3.72	78.24
14	R6	0	0	0	3.68	7.73	72.08
13	R7	0	0	0	3.54	8.67	73.13
8	R8	1	0	1	1.35	3.86	90.18
7	R9	−1	0	1	1.42	3.25	69.05
10	R10	0	1	−1	6.74	13.24	70.14
3	R11	−1	1	0	4.26	8.36	60.17
17	R12	0	0	0	4.25	7.49	75.58
2	R13	1	−1	0	3.14	7.35	92.26
1	R14	−1	−1	0	3.28	8.43	78.54
9	R15	0	−1	−1	6.52	12.65	77.85
16	R16	0	0	0	3.86	9.75	73.31
5	R17	−1	0	−1	6.84	11.72	69.14

**Table 3:** Evaluation of blend uniformity of physical mixture and assay of pellets.

Formulation Code	Physical Mixture Blend Uniformity (%*w*/*w*)	Pellets Drug Content (Assay) (%*w*/*w*)
R1	99.2 ± 1.7	99.8 ± 2.3
R2	101.6 ± 2.4	101.2 ± 2.1
R3	101.4 ± 1.9	100.6 ± 1.4
R4	102.3 ± 2.1	104.7 ± 2.7
R5	101.9 ± 1.3	102.4 ± 1.8
R6	98.2 ± 1.6	98.6 ± 2.2
R7	100.3 ± 1.4	101.2 ± 2.1
R8	98.1 ± 2.3	100.2 ± 2.3
R9	101.2 ± 1.7	99.4 ± 1.6
R10	100.2 ± 2.3	102.7 ± 2.3
R11	99.7 ± 1.6	101.4 ± 1.5
R12	98.8 ± 1.5	99.6 ± 2.1
R13	101.2 ± 1.3	100.8 ± 2.4
R14	100.6 ± 2.2	101.3 ± 2.5
R15	102.4 ± 1.4	101.6 ± 1.4
R16	101.3 ± 2.5	102.4 ± 2.2
R17	99.8 ± 1.8	98.3 ± 1.3

**Table 4:** *p*-value and estimated coefficients of the regression models for the studied responses.

Source	Y1: Q2			Y2: Q5			Y3: Q24		
	*f*-Value	*p*-Value	Coefficient Estimate	*f*-Value	*p*-Value	Coefficient Estimate	*f*-Value	*p*-Value	Coefficient Estimate
Model	516.95	<0.0001		321.89	<0.0001		71.49	<0.0001	
Intercept			3.87			8.14			74.06
A—Pectin LM							146.24	<0.0001	9.61
B—HPMC HME 4M							49.12	<0.0001	−5.57
C—Eudragit^®^ S 100	516.95	<0.0001	−2.67	321.89	<0.0001	−4.41			
AB									
AC									
BC									
A^2^							19.11	0.0008	4.78
B^2^									
C^2^									
Lack of Fit	1.36	0.4130		0.4262	0.8813		3.15	0.1403	

Model *p* < 0.05: Statistically significant; Lack of Fit *p* > 0.05: Lack of Fit is not significant.

**Table 5:** Model Validation by Comparison of the Predicted and Observed Experimental Values.

Checkpoints	Independent Variables			Responses								
	Factor A:	Factor B:	Factor C:	Y1: Q2 (%)			Y2: Q5 (%)			Y3: Q24 (%)		
	Pectin LM (mg)	HPMC HME 4M (mg)	Eudragit^®^ S 100	Predicted Value (%)	Experimental Value (%)	Standard Error	Predicted Value (%)	Experimental Value (%)	Standard Error	Predicted Value (%)	Experimental Value (%)	Standard Error
R9	50	125	15	1.20	1.42	0.143	3.73	3.25	0.298	69.23	69.05	1.124
R13	100	100	10	3.87	3.14	0.081	8.14	7.35	0.169	94.02	92.26	1.377
R15	75	100	5	6.55	6.52	0.143	12.58	12.65	0.298	79.63	77.85	1.092

## Data Availability

The data presented in this study are available within the article.
